# Engaged teachers and well-being: the mediating role of burnout dimensions

**DOI:** 10.1080/21642850.2024.2404507

**Published:** 2024-10-03

**Authors:** Giacomo Angelini, Caterina Mamprin, Ivan Borrelli, Paolo Emilio Santoro, Maria Rosaria Gualano, Umberto Moscato, Caterina Fiorilli

**Affiliations:** a Department of Human Sciences, University of LUMSA, Rome, Italy; b Faculté des sciences de l’éducation, Département de psychopédagogie et d’andragogie, Université de Montréal, Montréal, Canada; c Department of Life Sciences and Public Health, Catholic University of the Sacred Heart, Rome, Italy; d Department of Women, Children and Public Health Sciences, Fondazione Policlinico Universitario Agostino Gemelli IRCCS, Rome, Italy; e School of Medicine, Saint Camillus International University of Health Sciences, UniCamillus, Rome, Italy

**Keywords:** burnout, teacher, well-being, mediation model, BAT, engagement

## Abstract

**Background:**

Engaged teachers experience a positive, fulfilling, and work-related state of mind related to their work tasks able to affect their well-being positively. Nevertheless, teachers are particularly exposed to burnout risk, which is highly probable to occur during teachers’ professional careers. The current study investigates the mediating effect of burnout, through which work engagement influences subjective well-being.

**Methods:**

Participants were 807 Italian teachers (Female, 91.7%; *M_age _*= 47.54; SD = 9.91). Self-report instruments were administered to evaluate teachers’ burnout (BAT, Burnout Assessment Tool), well-being (WHO-5 Well-being Index), and work engagement (UWES-3, Utrecht Work Engagement Scale).

**Results:**

Findings show that exhaustion (*β *= −0.2162, *p *< 0.001) and psychological distress (*β *=* *−0.2811, *p *< 0.001) mediate the relationship between work engagement and well-being (total effect, *β *= 0.6409, *p *< 0.001).

**Conclusions:**

These results enable us to gain a deeper understanding of how the phenomenon of burnout impacts teachers’ well-being, allowing us to design training, prevention, and evaluation programs that consider the complex nature of burnout.

## 1. Introduction

Teaching is a helping profession based on caring (i.e., toward students) and collaborative relationships (i.e., with colleagues and school leaders). Consequently, during their career, teachers may experience both enthusiasm and motivation in their work, as well as fatigue due to high emotional demand (Kariou et al., 2021; Kinman et al., 2011; Lazarides et al., 2020). Therefore, the balance between personal resources and job demands is crucial for teachers’ well-being (Bakker & Demerouti, 2007). There is a strict relationship between work engagement (WE) and subjective well-being (SWB): the more teachers are engaged in their work, the more they are satisfied with their lives, which, in turn, improves SWB (Cho, 2021; Rusu & Colomeischi, 2020). In other words, WE has proven to predict SWB (Adil & Kamal, 2019; Shimazu et al., 2012), even considering findings from longitudinal studies (Hakanen & Schaufeli, 2012).

Nevertheless, teaching is a profession at high risk of burnout due to long-term stressful events that may occur during a professional career (Brady et al., 2023; Cacciamani et al., 2022; Fiorilli et al., 2017). Indeed, teachers tend to be more engaged at the beginning of their careers compared to the end, primarily due to the effects of burnout (Travers, 2017). Thus, the current study aims to analyze how and to what extent teachers’ burnout experience mediates the relationship between WE and SWB. Specifically, this study adopts a multi-component perspective, based on the recent advancements in measuring burnout as a complex construct (Schaufeli et al., 2020), to explore the distinct role of its dimensions: exhaustion, mental distance, cognitive impairment, emotional impairment, psychological distress, and psychosomatic complaints.

### 1.2. Theoretical framework

#### 1.2.1. Teachers’ work engagement and their well-being

WE is generally defined as a ‘positive, fulfilling, work-related state of mind characterized by vigor, dedication, and absorption’ (Schaufeli et al., 2002, p. 74). Research has shown that engaged workers are more resilient and persistent in dealing with hardship; they experience a sense of meaning, enthusiasm, inspiration, and pride in their work (Van Wingerden et al., 2017). Furthermore, they tend to approach tasks with full absorption and concentration (Bakker et al., 2014; Schaufeli et al., 2002). In the context of teaching, several positive outcomes have been observed in engaged teachers, including greater commitment to students’ achievement, curiosity and enthusiasm toward new learning opportunities for themselves, and persistent behavior in difficult situations (Burić & Macuka, 2018; Lauermann & König, 2016). The positive emotions strictly related to teachers’ WE support the Leiter and Bakker (2010) assumption that work engagement effectively is work-related well-being characterized by a positive affective-motivational state that impacts both individual and organizational outcomes (Bakker & Demerouti, 2008; Schaufeli et al., 2002).

Previous studies have effectively argued that WE is positively related to SWB (Bakker et al., 2014; Bauer et al., 2014; Hakanen & Schaufeli, 2012; Han et al., 2020; Perera et al., 2018; Yan et al., 2019), which is mainly defined as ‘a person's cognitive and affective evaluations of his or her life’ (Diener et al., 2002, p. 63). This concept refers to how people evaluate the internal experience of personal and social quality of life (Topp et al., 2015) based on the perception of general life satisfaction (Pawlowski et al., 2011) and the subjective assessment of one's current state in the world (Lopez et al., 2018). In recent years, teachers’ well-being has been investigated through two main conceptual frameworks. The eudaimonic perspective is associated with psychological and social functioning, reflecting psychological well-being (Chen et al., 2013; Delle Fave et al., 2011; Joshanloo, 2019; Samman, 2007). In contrast, the hedonic perspective pertains to positive effects and life satisfaction, commonly linked to subjective well-being (Deci & Ryan, 2008; Delle Fave et al., 2011; Joshanloo, 2019; Samman, 2007). Previous studies on teachers have demonstrated a relationship between WE and SWB, showing a permeability between school and private life. As a result, engaged teachers tend to feel more satisfied with their lives (Cameron & Lovett, 2015; Han et al., 2020; Perera et al., 2018). Recent studies show that a work environment in which people feel good contributes not only to higher job satisfaction and lower levels of burnout but also to general well-being (Toyama et al., 2022).

As we have seen, the link between teacher WE and teachers’ SWB is largely confirmed by the literature. However, despite this well-established association, work engagement is not in a stable psychological state. Rather, it fluctuates during teachers’ careers due to several sources of stress menacing their mental health.

#### 1.2.2. The mediating role of teachers’ burnout

According to recent studies by Schaufeli and colleagues (2017), burnout can be defined as ‘a work-related state of exhaustion that occurs among employees, which is characterized by extreme tiredness, reduced ability to regulate cognitive and emotional processes, and mental distancing. These four core dimensions of burnout are accompanied by depressed mood as well as by non-specific psychological and psychosomatic complaints’ (p. 4). When this phenomenon occurs in a school context, it is referred to as teacher burnout, an experience of physical and emotional fatigue characterized by an inability to meet job demands, a loss of pleasure in teaching, and a decreased engagement in one's profession (Kyriacou, 2015; Skaalvik & Skaalvik, 2018). Teacher burnout results from exposure to school-related stress factors and an imbalance between high job demand (e.g., work overload, emotional job demand, conflicts at work) and low personal resources to face prolonged labor challenges (e.g., social support resources, Arvidsson et al., 2019; Bakker & Demerouti, 2017; Kyriacou, 2015; Madigan et al., 2023). High burnout levels can negatively affect job performance, increasing the risk of absenteeism and turnover (Laybourn et al., 2019; Madigan & Kim, 2021).

Since the earliest studies on teachers’ burnout, scholars have employed the classic three-dimension measure, which included exhaustion, detachment, and reduced accomplishment (Maslach, 1998). However, the extensive body of research and rich findings over the years have led scholars to assume a broader perspective on burnout symptoms. According to Schaufeli and colleagues (2017), and summarizing several research findings (Oliveira et al., 2021), in addition to the aforementioned symptoms of exhaustion (chronic fatigue, physical, and mental loss of energy) and mental distance (feeling of cynicism, aversion to the job, and reduced interest and enthusiasm), burnout also affects emotion regulation, cognitive processes, psychology, and psychosomatic outcomes. Indeed, burnout affects several emotion regulation processes, causing emotional impairment, which in turn is the cause of the feeling of unmotivated sadness or inability to control negative emotions at work (Desart & De Witte, 2019).

Moreover, burned-out teachers may exhibit cognitive impairment, such as memory deficits and reduced attentional resources (Deligkaris et al., 2014). Research also demonstrated that burnout is often associated with psychological distress, such as insomnia, worries, and tension or anxiety. Finally, several psychosomatic complaints have been demonstrated to complement burnout, such as palpitations, chest pain, stomach, intestinal problems, headaches, or muscle pains (Desart & De Witte, 2019). Burnout symptoms, or the complete burnout syndrome, can be associated with WE in several ways. When teachers cultivate and maintain their WE, burnout risk decreases. Indeed, engagement helps teachers maintain their enthusiasm for teaching (Lizano et al., 2019) and allows them to react to school pressure work with greater resources and, therefore, better manage stressful situations (Benevene et al., 2018; Fiorilli et al., 2015). Effectively, WE has demonstrated an important role in countering the psychological distress of teachers (Passiatore et al., 2019; Pepe et al., 2021; Veronese & Pepe, 2014), positively influencing a series of psychosomatic disorders. On the other hand, when teachers experience high levels of burnout, this threatens their SWB. In particular, burnout has implications for well-being at work, teachers’ private lives, health, motivation, and job satisfaction (Sprang et al., 2007). Indeed, it cannot be thought that burnout affects only the working context of teachers but that it also has important repercussions in their personal and social lives (Bakker et al., 2009; González-Morales et al., 2012). The consequences of school burnout are not limited to the working environment alone but accompany the teachers in their private lives (Fiorilli et al., 2015). Emotional exhaustion due to burnout can drain personal resources of time and energy, to be reinvested effectively in taking part in family life (Bakker et al., 2011), increasing family conflicts (Byron, 2005; Ilies et al., 2015). While exhaustion and psychological distress have been well-studied (Kristensen et al., 2005), other dimensions of burnout remains less explored.

### 1.3. Aims and hypotheses

Based on the theoretical framework described, the current study aims to deeply analyze how and to what extent teachers’ burnout experience mediates the relationship between WE and SWB. Considering the wide dimensions involved in the burnout experience, we implemented a parallel mediation model to analyze the relationships between teachers’ WE and SWB, with multiple burnout dimensions acting as mediators. Nonetheless, due to the absence of previous studies specifically focused on multiple burnout dimensions, we formulated the following research question: how do the dimensions of burnout (i.e., exhaustion, mental distance, cognitive impairment, emotional impairment, psychological distress, and psychosomatic complaints) affect the relationship between WE and SWB within teachers’ samples?

Based on previous studies, we set the following hypotheses:

Hypothesis 1: All burnout dimensions (exhaustion, mental distance, cognitive impairment, emotional impairment, psychological distress, and psychosomatic complaints) were negatively correlated with both WE and SWB (H1);

Hypothesis 2: All burnout dimensions negatively mediated the relationship between WE and SWB (H2).

## 2. Method

### 2.1. Participants

Participants were 807 Italian teachers in primary (65.3%) and high schools (34.7%) (age range = 19–68, *M_age _*= 47.54, *SD* = 9.91), 740 of whom were women (91.7%), and 67 were men (8.3%). The criteria for inclusion in the study were that the participants were Italian teachers and voluntarily agreed to participate.

### 2.2. Data collection and procedure

The current study was conducted in Italy, adopting a cross-sectional descriptive design and using convenience sampling. The data was collected via an online survey administered on Google Forms, which took approximately 15 minutes to complete. Before completing the survey, participants were informed of the research objectives and informed consent for procedures for gathering and processing data. The data was collected anonymously; participants voluntarily joined, freely and without receiving compensation. No data were excluded, and no missing data was found. This study was conducted under the privacy and informed consent requirements laid down by current Italian law (Law Decree DL-196/2003). The research project was accepted by the Ethics Committee for Scientific Research (CERS) of LUMSA University, and the study was conducted under the Declaration of Helsinki.

### 2.3. Instrumentation

#### 2.3.1. Burnout assessment tool

The Burnout Assessment Tool (BAT, Schaufeli et al., 2020; Italian version, Angelini et al., 2021) consists of the BAT-C and BAT-S. The BAT-C includes 23 items, measuring four core dimensions of burnout: Exhaustion (E), Mental distance (MD), Cognitive impairment (CI), and Emotional impairment (EI). The BAT-S includes ten items measuring secondary symptoms: Psychological distress (PD) and Psychosomatic complaints (PC). All items were scored on a five-point Likert scale, ranging from *‘never’* (1) to *‘always’* (5). Responses were summed and averaged for each subscale; scoring ranged between one and five. Total BAT scores can assess burnout, while independent scores on their six dimensions (core and secondary symptoms) can provide more information on teachers’ burnout as a complex phenomenon that extends beyond mere exhaustion and is influenced by several factors. BAT has demonstrated satisfactory psychometric properties with adequate internal consistency and excellent Cronbach's α (*α* = 0.933) and McDonald’s ω (*ω* = 0.934).

#### 2.3.2. World Health Organization Well-being Index

The World Health Organization's Five Well-being Index (WHO-5; Bech, 2004; Italian version, Topp et al., 2015) is a self-report questionnaire that evaluates global well-being. The WHO-5 comprises five items rated on a six-point Likert scale, from *‘all of the time’* (0) to *‘at no time’* (5). In the current study, WHO-5 has demonstrated satisfactory psychometric properties, with adequate internal consistency and good Cronbach's α (*α* = 0.888) and McDonald’s ω (*ω* = 0.889).

#### 2.3.3. Utrecht Work Engagement Scale

The Utrecht Work Engagement Scale (UWES-3; Schaufeli et al., 2017; Italian version, Balducci et al., 2010) is a short scale composed of 3 items, that measures work engagement and evaluates its three dimensions: Vigor, Dedication, and Absorption. Each item is rated on a seven-point Likert scale from *‘never’* (0) to *‘always’* (6). Alphas were acceptable (>0.70) in five national samples of the original study. In the current study, UWES-3 has demonstrated satisfactory psychometric properties, with adequate internal consistency and good Cronbach's α (*α* = 0.834) and McDonald’s ω (*ω* = 0.840).

#### 2.3.4. Socio-demographic information

A series of questions were also administered that investigated the socio-demographic field, i.e., gender, age, and education level.

### 2.4. Ethics statement

- Institutional Review Board Statement: The study was conducted in accordance with the Declaration of Helsinki and was approved by an Institutional Review Board/Ethics committee. See details under Methods.
- The study received an exemption from an Institutional Review Board/Ethics committee. See details under Methods.

## 3. Analysis

**Figure 1:**
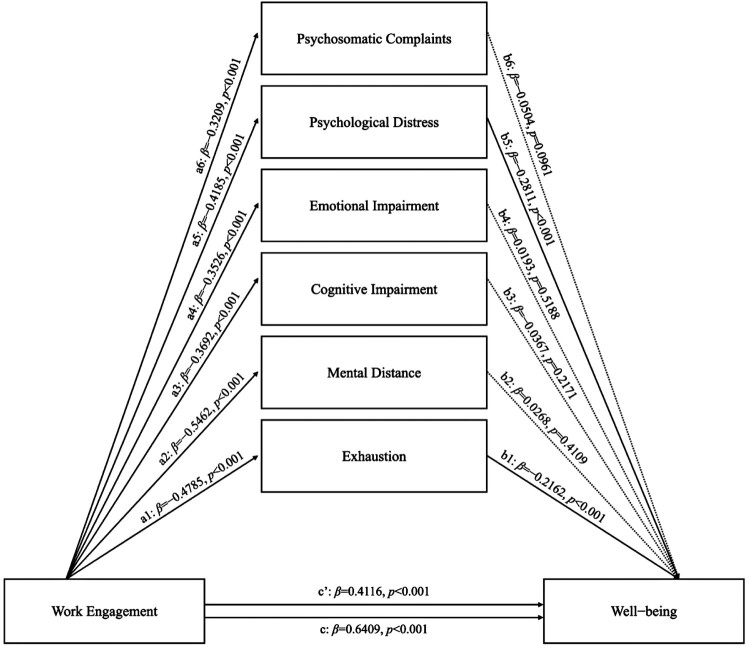
The relationship between engagement and well-being, with different dimensions of burnout as parallel mediators: A parallel mediation model.

The SPSS statistical software (v.27.0 for Macintosh) was used to analyze the collected data. The means and standard deviations for all scales were calculated. The Kolmogorov–Smirnov test was conducted to verify the normality of the distribution. The *p*-value <0.01 was considered a significant deviation from the normality of the distribution. Spearman's correlation analysis evaluated the associations between the variables under study (burnout, engagement, and well-being). Spearman's correlation was preferred over Pearson's correlation because, in the presence of non-normal distributions – common in the assessment of burnout, well-being, and related concepts – Pearson's correlation can inflate Type I error rates and reduce statistical power (Bishara & Hittner, 2012). To evaluate the effect of engagement on well-being and explore the role of different dimensions of burnout, a parallel mediation model using the macro-program PROCESS 4.0 (Hayes, 2018) was tested. Concerning the model specification, WE was the predictor, SWB was the outcome, and the six dimensions of burnout were the mediator variables. The hypothesized model is shown in Figure 1. Specifically, Figure 1 shows the effect of WE on Exhaustion (a1), Mental distance (a2), Emotional impairment (a3), Cognitive impairment (a4), Psychological distress (a5), and Psychosomatic complaints (a6). Furthermore, Figure 1 shows the effect of Exhaustion (b1), Mental distance (b2), Cognitive impairment (b3), Emotional impairment (b4), Psychological distress (b5), and Psychosomatic complaints (b6) on well-being. Finally, Figure 1 also shows the total effect of WE on SBW (c) and the direct effect of WE on SWB (c′). The 95% confidence interval (CI) was calculated for each regression coefficient included in the models. Finally, the statistical relevance of the indirect effects was verified by performing the bootstrap technique for each of the 5000 bootstrapped samples within 95% of the confidence interval.

## 4. Results

Table 1 shows the measures’ means (*M*), standard deviations (*SD*), and Spearman's correlation matrix.

**Table 1.** Correlations between the dimensions of BAT, engagement, and teachers’ well-being.

	Mean	*SD*	BAT (total score)	Exhaustion	Mental Distance	Cognitive Impairment	Emotional Impairment	Psychological Distress	Psychosomatic Complaints	Well-being
BAT (total score)	1.947	0.506	–							
Exhaustion	2.414	0.746	0.855**	–						
Mental Distance	1.531	0.500	0.708**	0.592**	–					
Cognitive Impairment	1.581	0.572	0.666**	0.474**	0.493**	–				
Emotional Impairment	1.563	0.537	0.629**	0.388**	0.486**	0.523**	–			
Psychological Distress	2.282	0.808	0.829**	0.614**	0.476**	0.455**	0.471**	–		
Psychosomatic Complaints	2.031	0.706	0.690**	0.462**	0.350**	0.333**	0.333**	0.620**	–	
Well-being	16.368	4.803	−0.669**	−0.593**	−0.493**	−0.424**	−0.363**	−0.598**	−0.465**	–
Engagement	14.871	3.072	−0.555**	−0.502**	−0.594**	−0.404**	−0.345**	−0.423**	−0.338**	0.657**

Note. ** Correlation is significant at the 0.01 level (2-tailed). BAT=Burnout Assessment Tool; *SD*=Standard Deviation.

Results highlighted that SWB and WE were significant and positively correlated with each other (*r* = 0.641, *p* < 0.001). Furthermore, there were significant and negative associations of WE with Exhaustion (*r *= −0.478, *p* < 0.001), Mental distance (*r *= −0.546, *p* < 0.001), Cognitive impairment (*r *= −0.369, *p* < 0.001), Emotional impairment (*r *= −0.353, *p* < 0.001), Psychological distress (*r *= −0.418, *p* < 0.001), and Psychosomatic complaints (*r *= −0.321, *p* < 0.001). Similarly, there was a significant and negative correlation between SWB and all dimensions of Burnout, i.e., Exhaustion (*r *= −0.607, *p *< 0.001), Mental distance (*r *= −0.494, *p *< 0.001), Cognitive impairment (*r *= −0.422, *p *< 0.001), Emotional impairment (*r *= −0.385, *p *< 0.001), Psychological distress (*r *= −0.615, *p *< 0.001), and Psychosomatic complaints (*r *= −0.466, *p *< 0.001). These results partially support Hypothesis 1.

A parallel mediation was performed to investigate the mediator role of the different dimensions of burnout in the relationship between WE and SWB (see Table 2). The results showed that WE showed a significant and positive total effect on SWB (path c; *β *= 0.6409, *p *< 0.001; LLCI = 0.9190; ULCI = 1.0851). Specifically, WE had significant negative effects on Exhaustion (path a1; *β *= −0.4785, *p *< 0.001; LLCI* *= −0.1310; ULCI* *= −0.1015), Mental distance (path a2; *β *= −0.5462, *p *< 0.001; LLCI* *= −0.09833; ULCI* *= −0.0794), Cognitive impairment (path a3; *β *= −0.3692, *p *< 0.001; LLCI* *= −0.0807; ULCI* *= −0.0567), Emotional impaired (path a4; *β *= −0.3526, *p *< 0.001; LLCI* *= −0.0730; ULCI* *= −0.0503), Psychological distress (path a5; *β *= −0.4185, *p *< 0.001; LLCI* *= −0.1266; ULCI* *= −0.0935), and Psychosomatic complaints (path a6; *β *= −0.3209, *p *< 0.001; LLCI* *= −0.0887; ULCI* *= −0.0586). In turn, the Exhaustion was negatively and significantly related to WE (path b1; *β *= −0.2162, *p *< 0.001; LLCI* *= −1.8104; ULCI* *= −0.9714), as well as the psychological distress (path b5; *β *= −0.2811, *p *< 0.001; LLCI* *= −2.0874; ULCI* *= −1.2548). Entering the six burnout dimensions in the model parallelly, Exhaustion and Psychological distress played a significant role in the relationship between WE and SWB, albeit remaining significant after controlling the mediators (path c’; *β *= 0.4116, *p *< 0.001; LLCI = 0.5572; ULCI = 0.7298). Therefore, a partial mediation occurred (*R^2 ^*= 0.4108 *F*_(1, 805) _= 561.2213, *p *< 0.001). The bootstrap procedure confirmed the statistical relevance of this indirect effect (BootLLCI = 0.5307; BootULCI = 0.7558). These results partially support Hypothesis 2. The coefficients of the significant parallel mediation models are summarised in Table 2.

**Table 2.** Coefficients of the mediation models.

*Ant.*		Exhaustion				Mental Distance				Cognitive Impairment				Emotional Impairment		
		Coef.	SE	*p*		Coef.	SE	*p*		Coef.	SE	*p*		Coef.	SE	*p*
Engagement	a^1^	−0.116	0.008	<0.001	a^2^	−0.089	0.005	<0.001	a^3^	−0.069	0.006	<0.001	a^4^	−0.062	0.006	<0.001
Exhaustion		–	–	–		–	–	–		–	–	–		–	–	–
Mental Distance		–	–	–		–	–	–		–	–	–		–	–	–
Cognitive Impairment		–	–	–		–	–	–		–	–	–		–	–	–
Emotional Impairment		–	–	–		–	–	–		–	–	–		–	–	–
Psychological Distress		–	–	–		–	–	–		–	–	–		–	–	–
Psychosomatic Complaints		–	–	–		–	–	–		–	–	–		–	–	–
*Costant*	*i_M_*_1_	4.143	0.114	<0.001	*i_M_*_2_	2.853	0.073	<0.001	*i_M_*_3_	2.603	0.093	<0.001	*i_M_*_4_	2.480	0.088	<0.001
		*R*^2^= 0.229				*R*^2^= 0.298				*R*^2^= 0.136				*R*^2^= 0.124		
		*F*_(1, 805) _= 239.0154, *p *= <0.001				*F*_(1, 805) _= 342.2272, *p *= <0.001				*F*_(1, 805) _= 127.0741, *p *= <0.001				*F*_(1, 805) _= 114.2771, *p *= <0.001		

*Ant.*		Psychological Distress				Psychosomatic Complaints				Well-being		
		Coef.	SE	*p*		Coef.	SE	*p*		Coef.	SE	*p*
Engagement	a^5^	−0.110	0.008	<0.001	a^6^	−0.074	0.008	<0.001	c'	0.644	0.044	<0.001
Exhaustion		–	–	–		–	–	–	b^1^	−1.391	0.214	<0.001
Mental Distance		–	–	–		–	–	–	b^2^	0.257	0.313	0.411
Cognitive Impairment		–	–	–		–	–	–	b^3^	−0.308	0.249	0.217
Emotional Impairment		–	–	–		–	–	–	b^4^	0.173	0.268	0.519
Psychological Distress		–	–	–		–	–	–	b^5^	−1.671	0.212	<0.001
Psychosomatic Complaints		–	–	–		–	–	–	b^6^	−0.343	0.206	0.096
*Costant*	*i_M_*_5_	3.918	0.128	<0.001	*i_M_*_6_	3.127	0.116	<0.001	*i_y_*	14.489	1.044	<0.001
		*R*^2^= 0.175				*R*^2^= 0.103				*R*^2^= 0.586		
		*F*_(1, 805) _= 170.9162, *p *= <0.001				*F*_(1, 805) _= 92.3955, *p *= <0.001				*F*_(7, 799) _= 161.7272, *p *= <0.001		

*Note*. BAT=Burnout Assessment Tool.

## 5. Discussion

In recent years, daily challenges in teaching have increased (e.g., the use of technology, multicultural classrooms, students with special needs, and regulatory changes, OECD, 2018). This has led to a higher risk of absenteeism, turnover, and burnout in the workplace (Benevene et al., 2020) and can also negatively impact teachers' lives and SWB. In turn, this inevitably affects students’ academic adaptation and their learning processes, significantly reducing the quality of education (Arens & Morin, 2016; Fiorilli et al., 2020; Madigan & Kim, 2021). For this reason, research on the variables that influence teachers’ SWB is fundamental to reducing the risk of burnout and all its consequences (Sabagh et al., 2018).

According to the scientific literature, WE has both direct and indirect positive effects on the SWB of teachers (Bakker et al., 2014; Bauer et al., 2014; Hakanen & Schaufeli, 2012), students (Kelly, 2012; Romano et al., 2020; Romano et al., 2021a, 2021b, 2021c; Schenke et al., 2015; Shen et al., 2015), and organizations (Salanova et al., 2010). In the current study, we explored the role of burnout in mediating the relationship between WE and SWB. We still know little about this relationship, as measurements of burnout generally have focused on investigating the dimensions of emotional exhaustion, cynicism, and professional efficacy (Maslach & Jackson, 1981). Nevertheless, the classic three-dimensional structure of burnout is far removed from describing burnout as a complex phenomenon (Szigeti et al., 2017). The current study aims to overcome the gap by adopting the Burnout Assessment Tool (BAT, Schaufeli et al., 2020), offering a broader perspective on people’s burnout experience. In this regard, it became particularly valuable to examine the mediating role of each BAT’s dimensions, encompassing emotional, cognitive, and physical burnout symptoms.

As expected, our results supported Hypothesis 1, showing negative correlations between all burnout dimensions with both WE and SWB (H1). These result is in line with previous findings from national and international research, which has demonstrated that the higher levels of WE are associated with lower symptoms of burnout throughout one's career (Anthony-McMann et al., 2017; Carlsson et al., 2019; Fiorilli et al., 2017, 2019; Schaufeli et al., 2020). Specifically, WE was negatively correlated with various dimensions of burnout, such as exhaustion (Byrne et al., 2016; Chen et al., 2020; Hakanen et al., 2018), mental distance (Darling-Hammond et al., 2017; Fiorilli et al., 2019; Gorozidis & Papaioannou, 2014; Nguyen et al., 2018), cognitive impairment (Ceschi et al., 2017; Deligkaris et al., 2014; Elfering et al., 2011; Eskildsen et al., 2017; Fisher et al., 2017; Miranda et al., 2020), emotional impairment (Guarnaccia et al., 2018; Karatepe et al., 2020), psychological distress (Miranda et al., 2020; Hamilton Skurak et al., 2021), and psychosomatic complaints (Schaufeli & Bakker, 2004). Furthermore, our results revealed that higher levels of burnout were associated with lower levels of SWB, aligning with previous studies that have shown excessive work-related effort decreases teachers' SWB (De Stasio et al., 2017; Fiorilli et al., 2017, 2020). Similarly, the literature also reports a negative correlation between SWB and various burnout dimensions, including exhaustion (Capone & Petrillo, 2020; Dal Corso et al., 2020; Lizano, 2015), mental distance (Cadime et al., 2016; Lizano, 2015; Pagnin & de Queiroz, 2015), cognitive impairment (Stenfors et al., 2013), emotional impairment (Borrelli et al., 2022; Sakakibara et al., 2020), psychological distress (Benevene & Fiorilli, 2015), and psychosomatic complaints (Arpaci et al., 2020; Kang et al., 2020).

Regarding Hypothesis 2, we anticipated that the various dimensions of burnout would negatively mediate the relationship between WE and SWB. Given the scarcity of existing literature on this topic, we aimed to explore the specific mediating effects of these dimensions. Specifically, we examined how burnout dimensions, as measured by the BAT, influenced the relationship between WE and SWB in our sample of teachers (H2). The results partially support this hypothesis, showing that the burnout's mediating role was significant only for the dimensions of exhaustion and psychological distress. Notably, prior to the conceptualization of burnout as a multifaceted and complex construct, exhaustion and psychological distress were often considered the key dimensions of burnout (Gustafsson et al., 2011; Kristensen et al., 2005; Maslach et al., 2001). Previous research has emphasized that exhaustion can trigger other dimensions of burnout, such as mental distance (cynicism) and cognitive impairment (inefficacy; Lee & Ashforth, 1996; Leiter & Maslach, 1988). Exhaustion, which reflects emotional and physical depletion, can lead to detachment from work and a reduced sense of personal accomplishment (Maslach et al., 2001). Furthermore, psychological distress is closely correlated with burnout, as it encompasses symptoms of depression, anxiety, and negative attitudes (Angelini et al., 2024). This finding confirms the existing literature on the role of these burnout dimensions in mediating the relationship between WE and SWB, while also highlighting the importance of addressing the specific aspects of burnout to improve teachers’ well-being.

As highlighted in other studies, the effects of cognitive impairment and psychosomatic complaints were found to be statistically insignificant (Roemer, 2016). However, despite that result, both dimensions still indicated a negative trend in relation to SWB. This aligns with previous literature, which suggests that the repercussions of cognitive impairment (memory deficits, executive functions, and attention) and psychosomatic problems (cardiovascular health, chronic pain syndromes, back and neck problems, headaches, and chronic pain) can negatively impact teachers’ SWB (Arpaci et al., 2020; Benevene & Fiorilli, 2015; Capone & Petrillo, 2020; Kang et al., 2020). These factors may also contribute to an increase in depressive symptoms (Bianchi et al., 2022a, 2022b; Bianchi & Schonfeld, 2022; Sowden et al., 2022).

Even the dimensions of mental distance and emotional impairment, although not statistically significant, offer us valuable insights that can explain their positive trend on well-being. Our findings could be interpreted by the fact that mental distance may function as an adaptive coping mechanism in response to feelings of exhaustion caused by intense emotional demands at work (Maslach et al., 2001).

On the other hand, emotional impairment could have a positive relationship with well-being by acting as a protective factor against burnout. In fact, according to the process model of emotion regulation (Gross, 2002), emotion suppression is one of the two key strategies for regulating emotions. While emotion suppression, specifically the suppression of emotional expression, is generally viewed as a maladaptive response-focused strategy (Chen et al., 2016; Chen et al., 2019), often associated to psychological distress and psychopathology symptoms (Aldao et al., 2010; Hom et al., 2016), our data suggest it could have a positive effect on SWB. Indeed, while low WE may cause cynicism, high WE may facilitate exhaustion (Winefield, 2003). A balance between these two levels can be achieved in terms of ‘emotionally detached concern’, a concept well-known in the medical field (Dormann & Zapf, 2004). This refers to physicians engaged in difficult diagnoses and invasive, sometimes noxious treatments aim to protect themselves from burnout and compassion fatigue by emotionally detached from patients (Sturzu et al., 2019). This principle could also apply to other helping professionals, such as teachers, where detached concern could reduce the impact of stressors on teachers’ burnout and improve SWB.

## 6. Limitations

Despite the results being in line with the general literature, the limitations of this study need to be considered. First, the data are not generalizable, as the data collection was conducted online with a convenience sample where participating teachers joined voluntarily. In other words, it could limit the external validity of the research, as it may not represent the broader population.

Second, the sample was not gender-balanced to the detriment of the male gender. Although in Italy, teaching is a predominantly female occupation, and the percentage of males and females in our sample reflects that of teachers in Italy (OECD, 2018), the results might not be entirely generalizable to more heterogeneous samples in terms of gender. Future research should replicate this type of study in a population with different socio-demographic characteristics to observe how the dimensions of BAT behave in a more balanced sample and to test its invariance by considering age and gender (De Beer et al., 2020). Third, a cross-sectional design did not return a cause–effect relationship for the observed relationships. Future research could overcome these limitations using a longitudinal design, a paper-pencil data collection, and a more gender-balanced sample.

Finally, self-report questionnaires are subject to recall bias and social response desirability.

## 7. Practical implications

The innovative contribution of this study concerns the evaluation of burnout with Burnout Assessment Tool. As a multidimensional and accessible instrument, BAT addresses the urgent need for new procedures and tools for burnout evaluation, offering significant advantages for educational environments (Hiver et al., 2022). Unlike previously utilized tools to investigate burnout (MBI, Maslach & Jackson, 1986; CBI, Kristensen et al., 2005), BAT combines different facets of burnout experience, providing a more articulated insight into the syndrome (Angelini et al., 2021). BAT allows us to evaluate burnout not as a people's connection with their profession (Maslach et al., 2017), but as a complex experience that simultaneously includes emotional, psychological, and psychosomatic dimensions. The results of this study have significant practical implications for educators and policymakers aiming to enhance teachers’ well-being. Notably, the discovery that exhaustion and psychological distress significantly mediate the relationship between work engagement and well-being suggests that targeted interventions aimed at reducing these specific dimensions of burnout could potentially improve teachers’ overall well-being.

Schools and educational institutions should consider implementing targeted programs such as stress management workshops, mindfulness training, and resilience-building activities that address exhaustion and psychological distress (De Carvalho et al., 2021; Fabbro et al., 2020). Moreover, these findings highlight the importance of fostering a supportive work environment that promotes work engagement while addressing the risk factors associated with burnout (Benevene et al., 2020). Practical steps could include providing regular professional development opportunities, creating a culture of open communication where teachers feel comfortable discussing their challenges, and ensuring manageable workloads to prevent excessive stress. Additionally, these insights can inform the design of pre-service and in-service training programs for teachers. Training modules that educate teachers on the signs of burnout, strategies for self-care, and ways to maintain a healthy work-life balance can be beneficial (Suldo et al., 2020). Schools can also establish peer support groups where teachers can share experiences and coping strategies. The BAT, as a comprehensive tool, could allow us to understand better how burnout affects SWB and structure training, prevention, and evaluation programs for teachers’ burnout. Finally, although this study focuses on teachers, the implications could extend to other helping professions, such as doctors, nurses, psychologists, and social workers. Future research could explore the applicability of these findings across different professional contexts to develop a broader understanding of burnout and well-being.

## 8. Conclusions

Over the years, research has investigated the relationship between burnout and personal resources (personality and engagement; Angelini, 2023; Christian et al., 2011) and their effects on well-being. In recent years, research has focused on the role that WE plays in predicting teachers’ SWB (Rusu & Colomeischi, 2020) and burnout (Schaufeli & Bakker, 2004; Høigaard et al., 2012), in addition to the role that the latter plays in relation to teachers’ SWB (Aparisi et al., 2019). Additionally, the studies on teachers’ SWB have notably increased over the past decades due to increased sick leave, absenteeism, turnover, and teacher abandonment (Benevene & Fiorilli, 2015). Other studies have instead focused on how physical and psychosomatic problems undermine the state of general well-being (Arpaci et al., 2020; Capone & Petrillo, 2020; Kang et al., 2020) and can increase depressive symptoms (Bianchi et al., 2022a, 2022b; Schonfeld & Bianchi, 2022; Sowden et al., 2022). This study offers a broader perspective on burnout's role in teachers’ work engagement and subjective well-being.

## Data Availability

The data presented in this study are available on request from the corresponding author.
